# Supplementary material for: Identification of a BTV-Strain-Specific Single Gene That Increases Culicoides Vector Infection Rate
Source: Viruses. 2021 Sep 7;13(9):1781. doi: 10.3390/v13091781 (PMC8472919; doi:10.3390/v13091781)
Supplement: Supplementary file 1 [file viruses-13-01781-s001.zip › viruses-1356271-supplementary.pdf]

Identification of a BTV-Strain-Specific Single Gene That Increases *Culicoides* Vector Infection Rate

Honorata M. Ropiak, Simon King, Marc Guimerà Busquets, Kerry Newbrook, Gillian D. Pullinger, Hannah Brown, John Flannery, Simon Gubbins, Carrie Batten, Paulina Rajko-Nenow *,† and Karin E. Darpel †

The Pirbright Institute, Ash Road, Pirbright, Woking, Surrey GU24 0NF, UK;
* Correspondence: paulina.rajko-nenow@pirbright.ac.uk
† These authors contributed equally to this work.

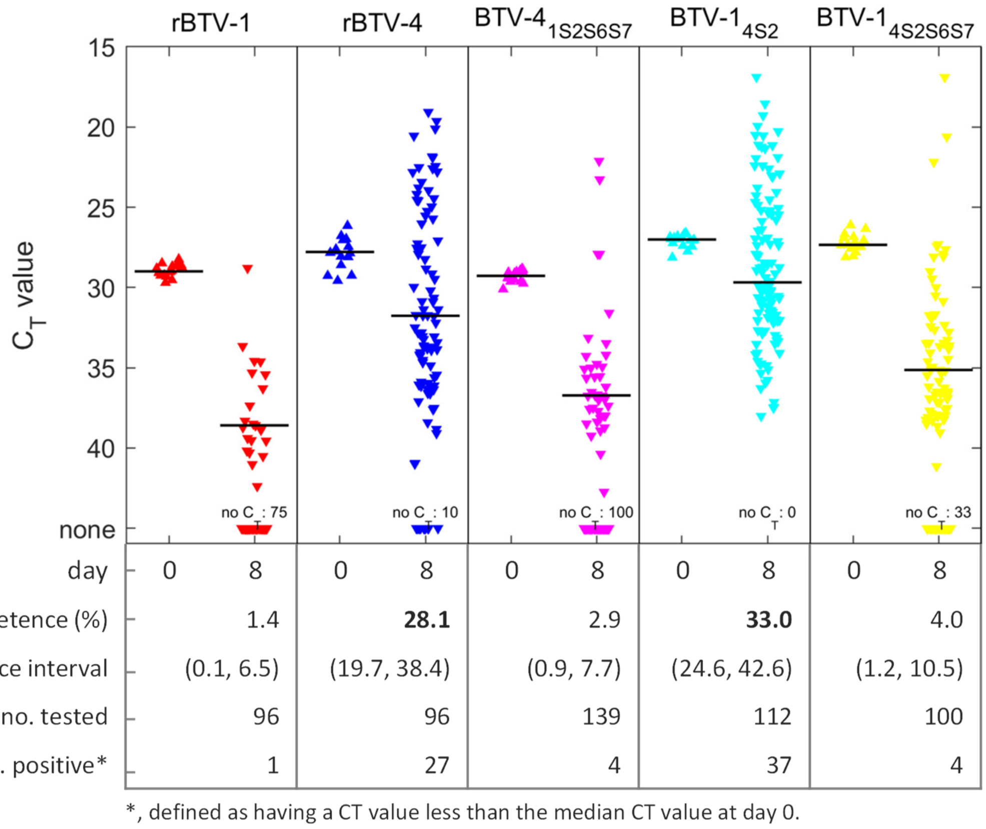

day	0	8	0	8	0	8	0	8	0	8
competence (%)		1.4		**28.1**		2.9		**33.0**		4.0
95% confidence interval		(0.1, 6.5)		(19.7, 38.4)		(0.9, 7.7)		(24.6, 42.6)		(1.2, 10.5)
no. tested		96		96		139		112		100
no. positive*		1		27		4		37		4

*, defined as having a CT value less than the median CT value at day 0.

Figure S1. Reverse-engineered and reassortant BTV strain C_T values in *C. sonorensis* after feeding. Individuals were tested either immediately after feeding (day 0) or after incubation for 8 days at 25 °C (day 8). Symbols depict the C_T values for individual insects and horizontal bars depict the median C_T value (excluding those individuals with no C_T value). C_T values were obtained from RT-qPCR assays of one independent experiment for each strain. Comparison amongst strains was carried out using a binomial-family generalized linear model with a logit link function. *Note:* Figure placed here for comparison to literature data, which is usually expressed in observed C_T values.

Table S1. Sequences of cloning primers. The upstream (forward) primers contained a T7 RNA polymerase promoter sequence and a restriction site for unidirectional cloning, while the downstream (reverse) primers contained restriction sites for cloning and for linearization at the 3' end of the segment [45].

BTV clone, segment	Sequence (5'-3')
**BTV-1 MOR2006/06**	
Seg-1 forward	TCTAGCGGATCCTAATACGACTCACTATAGTTAAAATGCAATGGTCGCAATC
Seg-1 reverse	TACAGTAAGCGGCCGCGTCTCAGTAAGTGTAATGCGGCGCGTGC
Seg-2 forward	TCTAGCGAATTCTAATACGACTCACTATAGTTAAAATAGTAGCGCGATGGATG
Seg-2 reverse	TACAGTAAGCGGCCGCGTCTCAGTAAGTCTAATAGTGCGCGGATC
Seg-3 forward	TCTAGCGAATTCTAATACGACTCACTATAGTTAAATTTCCGTAGCCATGGCTG
Seg-3 reverse	TACAGTAAGCGGCCGCGTCTCAGTAAGTGTGTTCCCGCTGCCGC
Seg-4 forward	TCTAGCGAATTCTAATACGACTCACTATAGTTAAAACATGCCTGAGCCACACG
Seg-4 reverse	CGTAAGCGGCCGCGGTCTCAGTAAGTTGTACATGCCCCCCTC
Seg-5 forward	TCTAGCGAATTCTAATACGACTCACTATAGTTAAAAAAGTTCTCTAGTTGGC
Seg-5 reverse	TACAGTAAGCGGCCGCGTCTCAGTAAGTTGAAAAGTTCTAGTAGAGTG
Seg-6 forward	CTAGCGAATTCTAATACGACTCACTATAGTTAAAAAGTGCACCCTTAGCGAA
Seg-6 reverse	TACAGTAAGCGGCCGCGGTCTCAGTAAGTGTAAGTGCTTCCCGTCGC
Seg-7 forward	TCTAGCGAATTCTAATACGACTCACTATAGTTAAAAATCTATAGAGATGGACA
Seg-7 reverse	TACAGTAAGCGGCCGCGGTCTCAGTAAGTGTAATCTAAGAGACGTTTG
Seg-8 forward	TCTAGCGGATCCTAATACGACTCACTATAGTTAAAAAATCCTTGAGTCATGGAG
Seg-8 reverse	CAGTAAGCGGCCGCGTCTCAGTAAGTGTAAATTCCCCCCCTAACC
Seg-9 forward	TCTAGCGAATTCTAATACGACTCACTATAGTTAAAAAATCGCATATGTCAGCTG
Seg-9 reverse	TACAGTAAGCGGCCGCGTCTCAGTAAGTGTAAAATCGCCCTACGTCA
Seg-10 forward	TCTAGCGAATTCTAATACGACTCACTATAGTTAAAAAGTGTCGCTGCCATGCT
Seg-10 reverse	TACAGTAAGCGGCCGCGTCTCAGTAAGTGTGTAGCGCCGCATACCCTC
**BTV-4 MOR2004/02**	
Seg-1 forward	GTGATGGATCCTAATACGACTCACTATAGTTAAAATGCAATGGTCGCAAT
Seg-1 reverse	GGTATGCGGCCGCGGTCTCAGTAAGTGTAATGCGGCGCGTG
Seg-2 forward	GTGATGAATTCTAATACGACTCACTATAGTTAAAAGAGTGTCCCACAATGG
Seg-2 reverse	GGTATGCGGCCGCGGTCTCAGTAAGTGTAAGAGGCCACAGG
Seg-3 forward	GTGATGAATTCTAATACGACTCACTATAGTTAAATTTCCGTAGCCATGG
Seg-3 reverse	GGTATGCGGCCGCCGTCTCAGTAAGTGTGTTCCCGCTGCCG

Seg-4 forward	GTGATGGATCCTAATACGACTCACTATAGTTAAAACATGCCTGAGCCACA
Seg-4 reverse	GGTATGCGGCCGCGGTCTCAGTAAGTTGTACATGCCCCCCT
Seg-5 forward	GTGATGAATTCTAATACGACTCACTATAGTTAAAAAAGTTCTCTAGTTG
Seg-5 reverse	GGTATGCGGCCGCCGTCTCAGTAAGTTGAAAAGTTCTAGTAG
Seg-6 forward	GTGATGAATTCTAATACGACTCACTATAGTTAAAAAGTGTTCTCCTACT
Seg-6 reverse	GGTATGCGGCCGCCGTCTCAGTAAGTGTAAGCTTCTCCCTCG
Seg-7 forward	GTGATGAATTCTAATACGACTCACTATAGTTAAAAATCTATAGAGATGG
Seg-7 reverse	GGTATGCGGCCGCGAAGACCAGTAAGTGTAATCTAAGAGACG
Seg-8 forward	GTGATGGATCCTAATACGACTCACTATAGTTAAAAAATCCTCGAGTCATG
Seg-8 reverse	GGTATGCGGCCGCCGTCTCA GTAAGTGTAAAATCCCCCCCT
Seg-9 forward	GTGATGAATTCTAATACGACTCACTATAGTTAAAAAATCGCATATGTCAG
Seg-9 reverse	GGTATGCGGCCGCCGTCTCAGTAAGTGTAAAATCGCCCTACG
Seg-10 forward	GTGATGAATTCTAATACGACTCACTATAGTTAAAAAGTGTCGCTGCCATG
Seg-10 reverse	GGTATGCGGCCGCCGTCTCAGTAAGTGTGTAGCGCCGCATA

Table S2. Restriction enzymes used for reverse genetics.

BTV clone, segment	Upstream site	Downstream site
**BTV-1 MOR2006/06**		
Seg-1	BamHI	BsmBI
Seg-2	EcoRI	BsmBI
Seg-3	EcoRI	BsmBI
Seg-4	BamHI	BsaI
Seg-5	EcoRI	BsmBI
Seg-6	EcoRI	BsaI
Seg-7	EcoRI	BsaI
Seg-8	BamHI	BsmBI
Seg-9	EcoRI	BsmBI
Seg-10	EcoRI	BsmBI
**BTV-4 MOR2004/02**		
Seg-1	BamHI	BsaI
Seg-2	EcoRI	BsaI
Seg-3	EcoRI	BsmBI
Seg-4	BamHI	BsaI
Seg-5	EcoRI	BsmBI
Seg-6	EcoRI	BsmBI
Seg-7	EcoRI	BbsI (BpiI)
Seg-8	BamHI	BsmBI
Seg-9	EcoRI	BsmBI
Seg-10	EcoRI	BsmBI

Table S3. Rescued and reassortant BTV strains [BSR3 passage] deposited in the Orbivirus Reference Collection (ORC) at the Pirbright Institute, UK (https://www.reoviridae.org/dsRNA_virus_proteins/ReoID/rescued%20BTV.html); where reverse engineered rBTV-1 is derivative of wtBTV-1 MOR2006/06 and rBTV-4 is derivative of wtBTV-4 MOR2004/02.

**ORC ref. number[a]**	**Virus name[b]**	**Genome segment(s)[c]**									
		**Seg-1**	**Seg-2**	**Seg-3**	**Seg-4**	**Seg-5**	**Seg-6**	**Seg-7**	**Seg-8**	**Seg-9**	**Seg-10**
BTV-RV0010*	rBTV-4										
BTV-RV0002	BTV-4_{1S3}			**1**							
BTV-RV0007	BTV-4_{1S9}									**1**	
BTV-RV0022*	BTV-$4_{1S2S6S7}$		**1**				**1**	**1**			
BTV-RV0042*	rBTV-1										
BTV-RV0021*	BTV-1_{4S2}		**4**								
BTV-RV0035	BTV-1_{4S3}			**4**							
BTV-RV0040	BTV-1_{4S9}									**4**	
BTV-RV0038*	BTV-$1_{4S2S6S7}$		**4**				**4**	**4**			

[a], all strains used in this study were after additional propagation to cell passage: BSR4.

[b], the first section of the name refers to the BTV backbone, with the second subscribed section referring to the heterologous segment(s). In this study, all strains were used after additional propagation to cell passage: BSR4

[c], white-boxes indicate those genome segments derived from wtBTV-1 MOR2006/06; grey-boxes indicate genome segments derived from wtBTV-4 MOR2004/02.

*, used in this study for in vivo oral infection of *C. sonorensis* experiments at cell passage: BSR4.

Table S4. Genome copy numbers obtained from BTV replication kinetics study for wild type (wtBTV-1 and wt-BTV-4) and reverse engineered (rBTV-1 and rBTV-4) strains.

dpi	wtBTV-1			wtBTV-4			rBTV-1			rBTV-4		
	mean gcn	sd	*n*	mean gcn	sd	*n*	mean gcn	sd	*n*	mean gcn	sd	*n*
0	$3.41×10^{2}$	$1.37×10^{2}$	6	$5.98×10^{3}$	$2.75×10^{3}$	6	$5.89×10^{2}$	$8.97×10^{2}$	6	$4.24×10^{2}$	$2.65×10^{2}$	12
1	$7.64×10^{2}$	$2.97×10^{2}$	6	$7.95×10^{3}$	$2.14×10^{3}$	6	$5.41×10^{2}$	$2.36×10^{2}$	6	$9.37×10^{2}$	$5.16×10^{2}$	12
2	$5.07×10^{3}$	$5.36×10^{3}$	6	$7.76×10^{3}$	$1.05×10^{3}$	6	$8.49×10^{2}$	$3.99×10^{2}$	6	$1.35×10^{3}$	$7.42×10^{2}$	12
3	$7.49x10^{4}$	$1.03×10^{5}$	6	$1.20×10^{4}$	$3.24×10^{3}$	6	$1.86×10^{3}$	$6.88×10^{2}$	6	$2.68×10^{3}$	$1.91×10^{3}$	12
6	$2.54×10^{6}$	$2.57×10^{6}$	6	$1.42×10^{6}$	$1.97×10^{6}$	6	$7.41×10^{5}$	$4.53×10^{5}$	6	$3.25×10^{5}$	$4.51×10^{5}$	12
7	$5.23×10^{6}$	$4.20×10^{6}$	6	$4.41×10^{6}$	$3.10×10^{6}$	6	$2.85×10^{6}$	$7.33×10^{5}$	6	$1.60×10^{6}$	$1.86×10^{6}$	12
10	$9.22×10^{6}$	$2.88×10^{6}$	6	$1.26×10^{7}$	$6.42×10^{6}$	6	$1.68×10^{7}$	$1.53×10^{7}$	6	$6.42×10^{6}$	$4.05×10^{6}$	12

dpi, days post infection; gcn, quantitated genome copy number per RT-qPCR reaction; sd, standard deviation; *n*, number of replicates (i.e. one independent experiment is composed of three replicates).

Table S5. Genome copy numbers obtained from BTV replication kinetics study for reverse engineered (rBTV-1 and rBTV-4) and reassortant (BTV-1_{4S2}, BTV-$1_{4S2S6S7}$ and BTV-$4_{1S2S6S7}$) strains.

dpi	BTV-1_{4S2}			BTV-$1_{4S2S6S7}$			BTV-$4_{1S2S6S7}$		
	mean gcn	sd	*n*	mean gcn	sd	*n*	mean gcn	sd	*n*
0	$1.50×10^{3}$	$8.20×10^{1}$	3	$5.00×10^{2}$	$2.75×10^{2}$	3	$3.09×10^{2}$	$1.31×10^{2}$	6
1	$2.02×10^{3}$	$4.27×10^{2}$	3	$1.09×10^{3}$	$2.05×10^{2}$	3	$6.82×10^{2}$	$1.96×10^{2}$	6
2	$2.89×10^{3}$	$1.99×10^{3}$	3	$1.19×10^{3}$	$9.70×10^{1}$	3	$2.23×10^{3}$	$5.65×10^{2}$	6
3	$1.38x10^{4}$	$6.00×10^{3}$	3	$5.77×10^{3}$	$3.40×10^{3}$	3	$8.99×10^{3}$	$5.63×10^{3}$	6
6	$1.04×10^{6}$	$4.03×10^{5}$	3	$5.64×10^{5}$	$1.87×10^{5}$	3	$6.17×10^{5}$	$6.79×10^{5}$	6
7	$2.72×10^{6}$	$6.14×10^{5}$	3	$1.39×10^{6}$	$1.92×10^{5}$	3	$1.80×10^{6}$	$9.68×10^{5}$	6
10	$7.03×10^{6}$	$1.73×10^{6}$	3	$4.10×10^{6}$	$2.34×10^{5}$	3	$3.68×10^{6}$	$1.93×10^{6}$	6

dpi, days post infection; gcn, quantitated genome copy number per RT-qPCR reaction; sd, standard deviation; *n*, number of replicates (i.e. one independent experiment is composed of three replicates).

Table S6. Deviance information criterion (DIC) for models assessing variation amongst BTV strains in replication curve parameters.

Model*				
**Replication rate**	**Asymptote**		**Initial no. copies**	**DIC†**
	**Lower**	**Upper**		
common	common	common	common	560.7
varies	common	common	common	474.6
common	varies	common	common	451.5
common	common	varies	common	531.2
common	common	common	varies	423.6
varies	varies	common	common	411.4
varies	common	varies	common	468.9
varies	common	common	varies	323.0
common	varies	varies	common	443.0
common	varies	common	varies	290.6
common	common	varies	varies	402.3
varies	varies	varies	common	411.9
**varies**	**varies**	**common**	**varies**	**251.2**
varies	common	varies	varies	317.5
common	varies	varies	varies	272.9
varies	varies	varies	varies	249.6‡

*, “common”: parameter common to all strains; “varies”: parameter varies amongst strains;
†, a model with a lower DIC is preferred to one with higher DIC;
‡, although this model has a smaller DIC than the one shown in bold, the difference is less than two and the simpler model was preferred as it has the smaller number of parameters.

Table S7. Comparison of posterior distributions of replication rates and time of maximum replication between BTV strains in KC cells.

Replication rate	Time of maximum replication
**wtBTV-4 > wtBTV-1 (P=0.03)**	**wtBTV-4 > wtBTV-1 (P<0.001)**
**rBTV-1 > wtBTV-1 (P=0.003)**	**rBTV-1 > wtBTV-1 (P<0.001)**
rBTV-4 = wtBTV-4 (P=0.06)	rBTV-4 = wtBTV-4 (P=0.78)
rBTV-1 = wtBTV-4 (P=0.55)	rBTV-1 = wtBTV-4 (P=0.20)
**rBTV-4 < rBTV-1 (P=0.01)**	**rBTV-4 > rBTV-1 (P=0.02)**
BTV-1$_{4S2}$ = rBTV-1 (P=0.05)	BTV-1$_{4S2}$ < rBTV-1 (P=0.05)
BTV-1$_{4S3}$ = rBTV-1 (P=0.60)	**BTV-1$_{4S3}$ < rBTV-1 (P<0.001)**
BTV-1$_{4S9}$ = rBTV-1 (P=0.39)	**BTV-1$_{4S9}$ < rBTV-1 (P<0.001)**
**BTV-1$_{4S2S6S7}$ < rBTV-1 (P=0.01)**	BTV-1$_{4S2S6S7}$ = rBTV-1 (P=0.16)
**BTV-4$_{1S2S6S7}$ < rBTV-1 (P<0.001)**	**BTV-4$_{1S2S6S7}$ < rBTV-1 (P=0.004)**
BTV-1$_{4S2}$ = rBTV-4 (P=0.53)	**BTV-1$_{4S2}$ < rBTV-4 (P=0.001)**
BTV-4$_{1S3}$ = rBTV-4 (P=0.50)	**BTV-4$_{1S3}$ < rBTV-4 (P<0.001)**
BTV-4$_{1S9}$ = rBTV-4 (P=0.13)	**BTV-4$_{1S9}$ < rBTV-4 (P=0.03)**
**BTV-4$_{1S2S6S7}$ < rBTV-4 (P=0.02)**	**BTV-4$_{1S2S6S7}$ < rBTV-4 (P<0.001)**
